# The effect of testing rate on biomechanical measurements related to stalk lodging

**DOI:** 10.1186/s13007-024-01253-9

**Published:** 2024-08-14

**Authors:** Kaitlin Tabaracci, Jacques Vos, Daniel J. Robertson

**Affiliations:** Department of Mechanical Engineering, University of Idaho, Moscow, ID 83844 USA

**Keywords:** Stalk, Lodging, Rate, Rind, Puncture, Bending, Stem, Strength, Stiffness

## Abstract

**Background:**

Stalk lodging (the premature breaking of plant stalks or stems prior to harvest) is a persistent agricultural problem that causes billions of dollars in lost yield every year. Three-point bending tests, and rind puncture tests are common biomechanical measurements utilized to investigate crops susceptibility to lodging. However, the effect of testing rate on these biomechanical measurements is not well understood. In general, biological specimens (including plant stems) are well known to exhibit viscoelastic mechanical properties, thus their mechanical response is dependent upon the rate at which they are deflected. However, there is very little information in the literature regarding the effect of testing rate (aka displacement rate) on flexural stiffness, bending strength and rind puncture measurements of plant stems.

**Results:**

Fully mature and senesced maize stems and wheat stems were tested in three-point bending at various rates. Maize stems were also subjected to rind penetration tests at various rates. Testing rate had a small effect on flexural stiffness and bending strength calculations obtained from three-point bending tests. Rind puncture measurements exhibited strong rate dependent effects. As puncture rate increased, puncture force decreased. This was unexpected as viscoelastic materials typically show an increase in resistive force when rate is increased.

**Conclusions:**

Testing rate influenced three-point bending test results and rind puncture measurements of fully mature and dry plant stems. In green stems these effects are expected to be even larger. When conducting biomechanical tests of plant stems it is important to utilize consistent span lengths and displacement rates within a study. Ideally samples should be tested at a rate similar to what they would experience in-vivo.

## Background

Stalk lodging (the permanent displacement of stalks/stems from their upright orientation) [1, 2] significantly reduces the yield of key crop species including maize and wheat. Yield losses due to stalk lodging are estimated to range from 5 to 20% annually [3, 4]. Stalk lodging occurs when externally applied forces (e.g., wind) create mechanical stresses within the plant that exceed the bending strength of the stem or stalk [5–8]. Given that maize and wheat are critical sources of food, feed and bioenergy [9] addressing the problem of stalk lodging is a key part of sustainably increasing agricultural outputs.

Stalk lodging resistance is a multifaceted agronomic trait that is influenced by spatially, and temporally varying factors at multiple length scales. Biomechanical tests of plant stalks are commonly used to gain insight into this complex biological phenomenon. For example, three-point bending tests have been used in many studies over the past century to determine the flexural stiffness and bending strength of plant stems [4, 10–17]. Both of these quantities are strongly related to lodging resistance [18]. Rind puncture tests have also been utilized by agronomists and plant science researchers for over 100 years [14, 19–35] as a rapid assessment of lodging resistance. However, researchers have come to conflicting conclusions regarding the efficacy of rind puncture resistance in determining stalk lodging resistance [18–20, 22, 23, 25, 34–36]. A recent study has demonstrated that while rind penetration resistance is a good predictor of the material properties of the rind tissue (e.g., Young’s Modulus) it is a poor predictor of structural bending properties of the entire stalk [37]. Furthermore, it was shown that rind puncture resistance measurements are only minimally influenced by key geometric determinants of lodging resistance (e.g., stalk diameter and rind thickness) [38, 39]. On the contrary, three-point bending tests and cantilever bending tests are strongly influenced by these key geometric determinants of stalk lodging.

Even though three-point bending and rind puncture tests are quite common, the effect of testing rate (i.e., strain rate) on rind puncture and three-point bending measurements of plant stems has not been directly investigated. Muliana et al. showed that hydrated sorghum stems exhibit time dependent mechanical properties [40]. In addition, it is well known among the human and animal biomechanics community as well as the wood science community that biological materials are generally rate dependent [41]. To effectively utilize rind penetration and three-point bending measurements to untangle the genetic determinants of stalk lodging resistance the effect of testing rate on such measurements needs to be quantified. If displacement rate has a significant influence on rind puncture and three-point bending measurements, then this would complicate large meta-analyses of multiple studies (that may have used different testing rates). Meta analyses are often required to draw firm conclusion regarding complex traits such as lodging resistance. In addition, if rate significantly affects three-point bending tests, then studies utilizing hand operated test stands instead of computer-controlled testing systems may be problematic because, rate cannot be precisely controlled in a hand operated test. Therefore, the purpose of this study was to evaluate the effect of testing rate (i.e., displacement rate) on rind puncture tests of maize and three-point bending tests of maize and wheat to develop best-practice guidelines.

## Methods

### Overview

Three-point bending experiments were performed on maize and wheat at multiple displacement rates to understand the effect of rate on flexural stiffness and bending strength measurements. Wheat samples were tested both with and without the leaf sheath as the leaf sheath has a significant effect on the biomechanical response of wheat stems [42]. Rind puncture experiments were performed on maize stalks at multiple displacement rates to understand the effect of displacement rate on puncture force and integrated puncture score measurements [35].

The displacement rates for each experiment were chosen based on practical limitations of standard laboratory equipment while simultaneously seeking to maintain data integrity by limiting experimental noise. Previous experimental studies [13, 17, 34, 35, 42] also informed rate selection. As it is common to have high samples sizes within plant-based experiments, very low displacement rates were excluded as these tests significantly lower throughput and can introduce viscoelastic effects (creep and stress relaxation). Additionally, very fast displacement rates were excluded to limit substantial inertial effects. It is also very difficult to maintain specimen orientation at higher displacement rates. Relatively slower displacement rates were used when testing wheat stems than maize stalks as wheat stems have smaller span lengths. Consequently, if tested at the same displacement rates as maize the strain rates would be much higher (see limitations section for a more in-depth discussion of strain rate).

### Plant materials

Maize stalks were collected from the University of Kentucky Spindletop Research Farm. All stalks were sourced from a single bag of Pioneer P1464AML seeds (mid-season, 114 days to mature). At harvest time (118 days after planting) stalk samples were collected by cutting each sample at the base and just above the primary ear bearing node. Leaves, ears and leaf sheaths were removed, and the stalks were spread in a single layer on wire rack benchtops in a greenhouse set to 36 °C with adequate air circulation to deter mold growth. Stalks remained in the greenhouse for one month after which they were transported to an airconditioned laboratory with a relative humidity that varies from 15 − 34% and an average temperature of 20 °C. Placing the stalks in the greenhouse and then in the lab allows long term storage of the stalks without degrading mechanical properties and closely resembles the condition of stalks in the field just prior to harvest [13, 16, 38, 43].

Wheat specimens were grown in Moscow, ID at University of Idaho Parker Farm. All samples were taken from the same plot of spring wheat. The stems were allowed to reach full maturity and remained in the field until harvest. The samples were cut at ground level and the most basal 30 centimeters of the plant was collected for testing. All samples were selected from the center of the plot to minimize border effects on stem growth [44]. Only samples that were found to be free of disease and in good mechanical condition were included in the study. All samples were stored in a laboratory space that is maintained at standard room temperature and humidity after collection (~ 20 °C and ~ 15-30% relative humidity). The wheat did not need to be stored in the greenhouse prior to being placed in the lab to prevent fungal growth. In addition, the leaf sheaths were not removed from the wheat plants prior to storing them in the laboratory as the leaf sheath significantly influences the bending stiffness and bending strength of wheat stems [42].

It is known among the biomechanics community that biological materials are generally rate dependent [40, 41]. One reason these materials display rate dependent properties is that they contain viscous fluids (e.g., water) which resist deformation and add material damping. As such, fully hydrated plant stems are expected to exhibit stronger rate dependent effects than dry plant samples. A deliberate choice was made to use fully mature and dry plant materials in this study because late season stalk lodging typically occurs near harvest time when the plants have senesced and dried down. Previous studies investigating late season stalk lodging have performed rind puncture and three-point bending tests of plant samples that were prepared in the same manner as those in this study. We were therefore also interested in what the effect of testing rate would be on stalks that had been prepared in this particular manner.

### Three-point bending tests of maize stalks

**Fig. 1:**
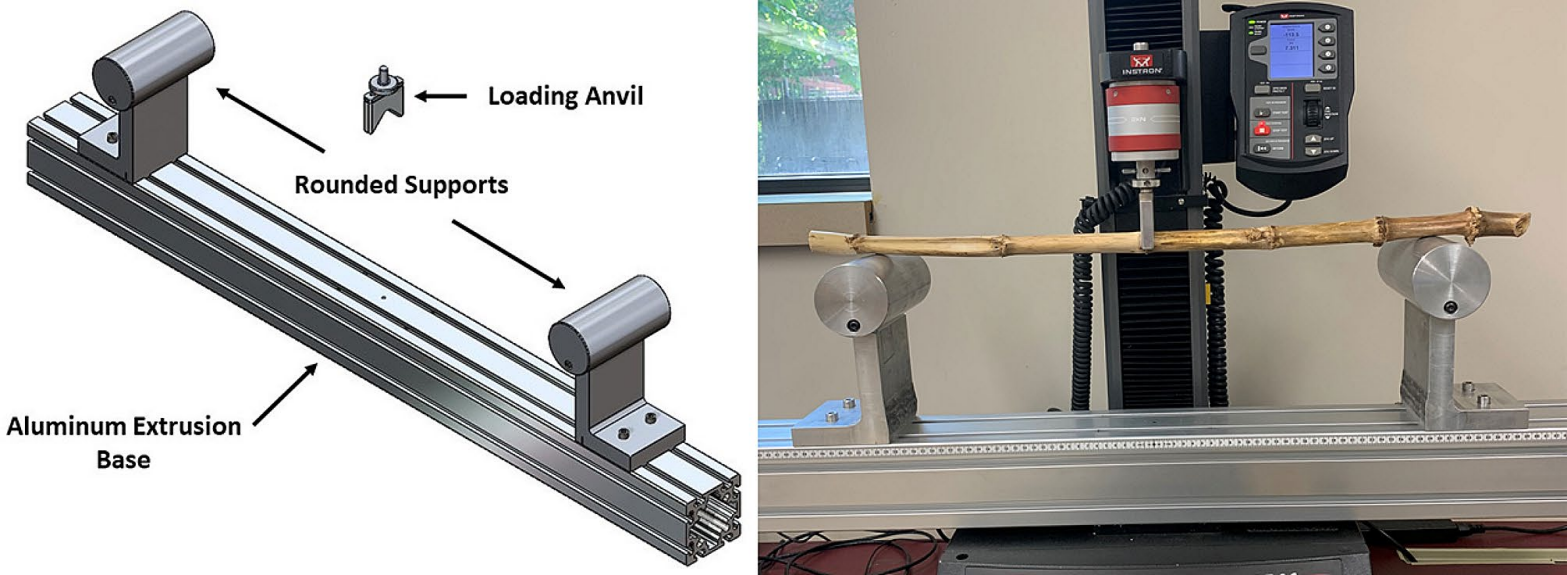
Custom long span three-point bending fixture [14]

**Fig. 2:**
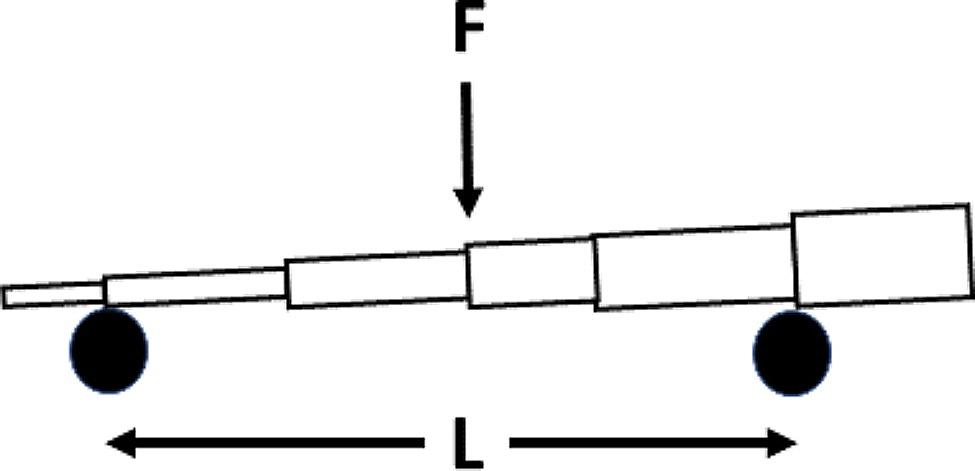
Diagram showing the experimental setup in terms of the variables used in Eqs. 1 and 2

Long span three-point bending tests were performed on a total of 110 maize stalk samples using a custom fixture that was mounted to a universal testing system. The base of the fixture was a 10.16-centimeters x 10.16-centimeters x 121.92-centimeters (4-inches x 4-inches x 48-inches) aluminum extrusion that had two L-shaped brackets approximately 15.24 centimeters (6 inches) tall affixed with 7.62-centimeter (3-inch) cylinders that supported the maize stalk samples during the test (Fig. 1). The loading anvil had an inverted “V” shape with rounded edges to prevent stress concentrations and premature collapse of the stalk cross-section that can occur when using a flat loading anvil [38]. A span length of 60 centimeters was used for all 110 maize stalk samples. Stalks were loaded at the center most node (halfway between the ear bearing node and the most basal node) as shown in Fig. 2. Stalks were loaded at the central most node as opposed to the center of an internode as this prevents crushing of the cross section and produces failure patterns similar to those observed for naturally lodged stalks [17, 45]. Maize stalks possess an elliptical cross-section; therefore, all stalks were loaded in the direction of the minor axis of the cross-section [14, 19, 35].

### Flexural stiffness calculations for maize stalks

A subset of 30 maize stalks were subjected to repeated, nondestructive three-point bending tests. Each stalk was tested at the seven displacement rates shown in Table 1. During each test the sample was preconditioned by displacing the stalk to 6 millimeters two times. The stalk was then displaced a third time to 6 millimeters and data from the last cycle of displacement was used to calculate stalk flexural stiffness (EI) using Eq. 1. Preconditioning is common best practice when testing biological specimens and is necessary to obtain repeatable force displacement responses [41]. For many biological specimens more than two cycles of preconditioning are required, however, preliminary testing of maize stalks in our lab revealed the force-displacement response of the stalk was repeatable after 2 cycles. All stalks were still within the elastic range after being displaced by 6 millimeters. In other words the stalks were not damaged or permanently deformed during any of these tests [16]. Thus, each stalk was able to be subjected to flexural test at each of the rates shown in Table 1, thereby enabling a paired statistical analysis of the results.

**Table 1:** Flexural stiffness was obtained from 30 maize samples at the following displacement rates

Rate 1	Rate 2	Rate 3	Rate 4	Rate 5	Rate 6	Rate 7
2.5 mm/min	5 mm/min	25 mm/min	100 mm/min	120 mm/min	240 mm/min	360 mm/min

Stalk flexural stiffness, *EI*, was calculated as follows [46]:

1 $$\:EI\:=\:\frac{\phi\:{L}^{3}}{48}$$

where ∅ is the slope of the force-displacement curve, acquired by flexing the stalk in three-point bending without plastically deforming the stalk and *L* is total the distance between the supports.

### Bending strength calculations of maize stalks

Characterizing the effect of displacement rate on stalk bending strength presents several challenges. Bending strength is a destructive measurement so each stalk can only be tested at a single rate, making repeated measurements of the same stalk at different rates impossible. Further compounding the issue is that no two maize stalks are exactly alike. Differences in stalk geometry among test specimens has a large effect on bending strength measurements and would likely obscure any differences in bending strength between groups tested at different displacement rates. However, it has been shown that flexural stiffness, which is a nondestructive measurement, is a very strong predictor of stalk bending strength. The R^2^ value between flexural stiffness and bending strength is frequently greater than 0.8 [16]. The authors utilized this correlation to investigate the effect of displacement rate on bending strength as explained below.

First each stalk was subjected to a nondestructive three-point bending test at a displacement rate of 100 mm/min following the flexural stiffness protocol outlined above. Data from this test was then utilized to calculate stalk flexural stiffness (at a rate of 100 mm/min). The strong linear correlation between stalk flexural stiffness and stalk bending strength was then utilized to predict the bending strength of the stalk (at a rate of 100 mm/min). After the nondestructive flexural stiffness test was complete, each stalk was subsequently tested to failure at either 2.5 mm/min, 25 mm/min, 100 mm/min or 360 mm/min. The actual bending strength at the tested rate was then compared to the predicted bending strength at a rate of 100 mm/min. A total of twenty stalks were tested to failure at each rate.

Bending strength was calculated using Eq. 2 [46]

2 $$\:{M}_{max}=\frac{FL}{4}$$

where *F* is the applied load and *L* is the distance between supports.

### Three-point bending tests of wheat stems

**Fig. 3:**
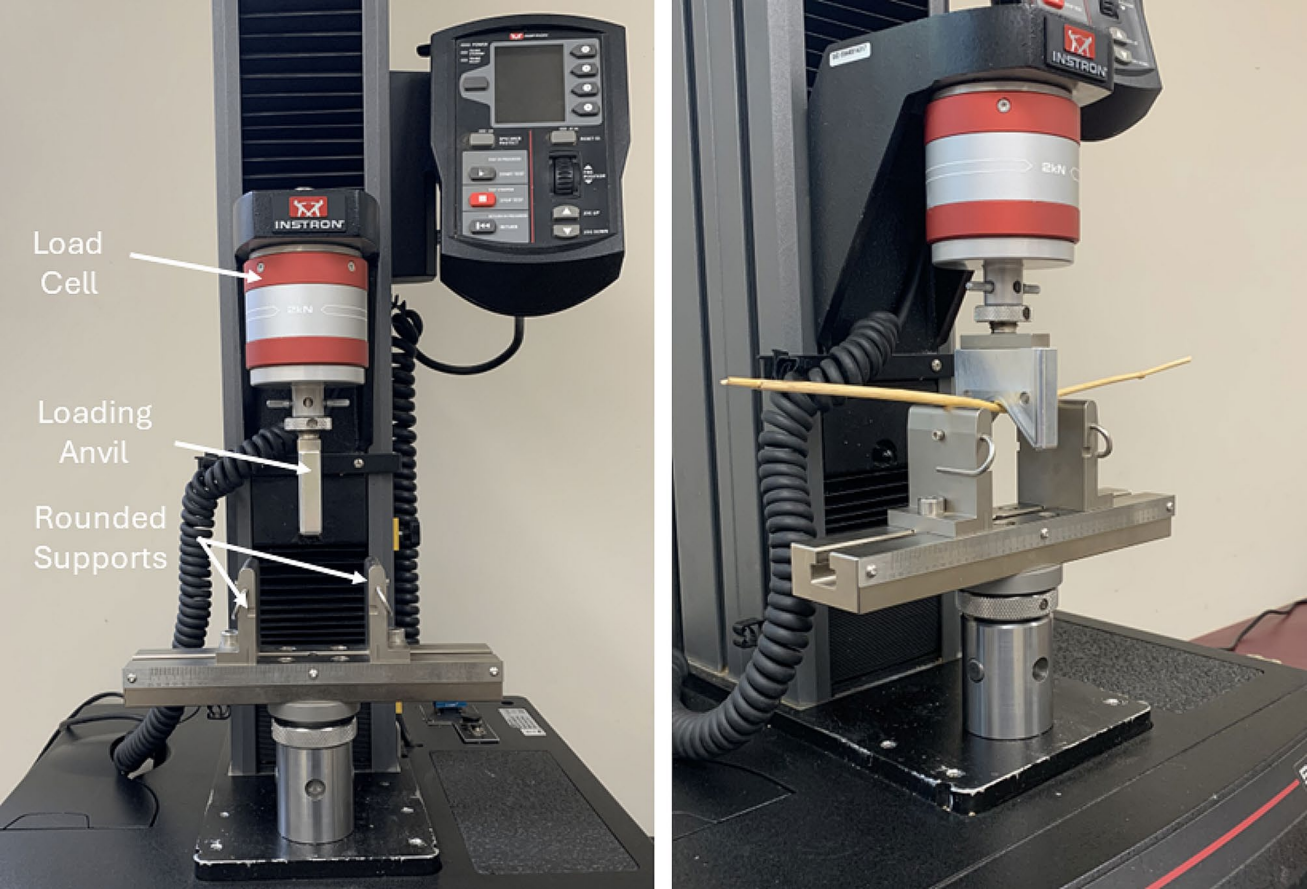
Three-point bending fixture for wheat stems

Three-point bending tests of wheat stems were conducted using an Instron 5kN flexure fixture (Part # 2810 − 400) with a span width of 8 centimeters (Fig. 3). The same general testing protocol for determining flexural stiffness of maize stalks was used for wheat stems. In particular, each stem was preconditioned by flexing it 2 times to a displacement of 1 mm. All stems remained within the elastic range after being displaced by 1 mm. Data from a third cycle of displacement was then used for flexural stiffness and bending strength calculations. Stems were tested at each of the rates seen in Table 2.

**Table 2:** Flexural stiffness was obtained from 60 wheat samples (30 with the leaf sheath and 30 without) at the following displacement rates

Rate 1	Rate 2	Rate 3	Rate 4	Rate 5	Rate 6	Rate 7
1 mm/min	2 mm/min	10 mm/min	40 mm/min	48 mm/min	96 mm/min	144 mm/min

### Flexural stiffness calculations for wheat stems

Flexural stiffness was calculated using Eq. 1. The leaf sheath of wheat stems has been shown to be an important structural feature of the plant [42]. Therefore, a total of 30 stems with the leaf sheath intact were tested at each of the rates shown in Table 2 to obtain flexural stiffness measurements. A second set of 30 stems that had the leaf sheath carefully removed prior to testing were also tested at each of the rates shown in Table 2. As flexural stiffness tests are nondestructive each of the stems were tested at each of the displacement rates displayed in Table 2, thereby enabling a paired statistical analysis of results.

### Bending strength calculations for wheat stems

To determine the effect of displacement rate on wheat stem bending strength the same general testing protocol used for maize was employed. In particular, each stem was first subjected to a nondestructive test at 40 mm/min to determine flexural stiffness. The flexural stiffness was then used to predict the bending strength of the stem at a rate of 40 mm/min. After the nondestructive flexural test each stem was subsequently tested to failure at either 1 mm/min, 40 mm/min or 144 mm/min. A total of 10 stems with the leaf sheath and 10 stems without a leaf sheath were displaced to failure at each rate. The bending strength of each stem at the tested rate was then compared to the predicted bending strength of that same stem at a rate of 40 mm/min.

### Rind puncture measurements of maize stalks

**Fig. 4:**
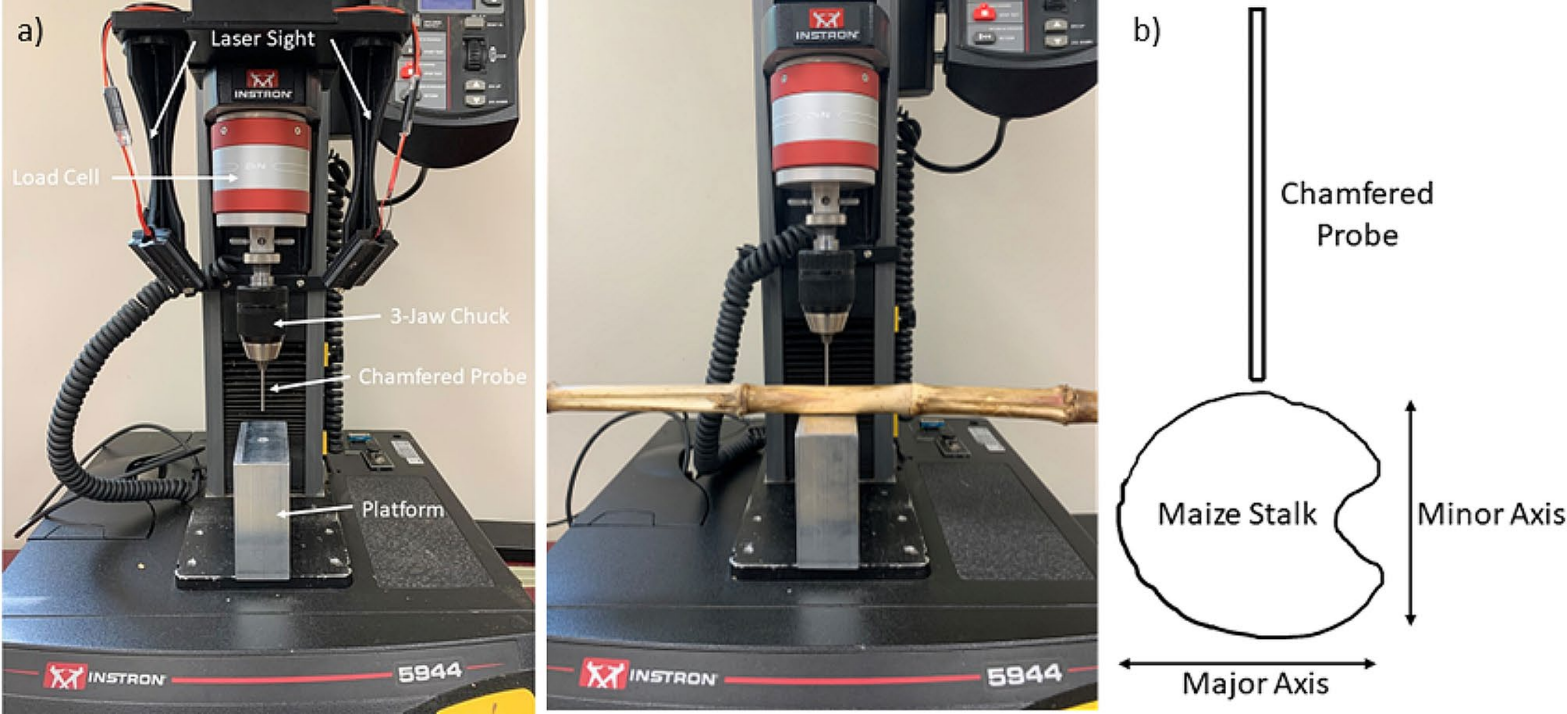
**(a)** Rind puncture experimental setup [14]. **(b)** Puncture diagram

Rind puncture tests were conducted using a Universal Testing machine equipped with a 2kN load cell. A custom aluminum platform was utilized that measures 4.445-centimeters x 10.16-centimeters x 10.16-centimeters (1.75-inches x 4-inches x 4-inches) with a 0.9525-centimeter (0.375-inch) hole in its center that allows for a chamfered puncture probe to pass completely through the stalk without impacting the platform. The chamfered puncture probe was 6.25 centimeters long, 2 millimeters in diameter with a 45-degree chamfer that reduced the diameter at the tip of the probe to 1 mm [34]. The test set up can be seen in Fig. 4a. Time, force and displacement data were collected at a rate of 1000 Hz. Maize stalks have an elliptical cross-section with the major axis of the ellipse typically being aligned with the ear groove. All stalks were punctured through the minor axis (see Fig. 4b) as this is standard practice and puncturing through ear groove can add experimental noise to measurements.

### Determining the effect of displacement rate on rind puncture

Like bending strength, rind puncture is a destructive measurement. Therefore, the same location on a stalk cannot be punctured at multiple different displacement rates. However, preliminary testing revealed that puncture tests occurring within a single internode produce nearly identical results. In particular, we punctured 12 maize internodes 4–5 times each at a rate of 25 mm/s. Each puncture test within a single internode occurred a minimum of 2.54 centimeters (1 inch) away from the site of any previous puncture tests and 2.54 centimeters (1 inch) away from the nodes. This preliminary data as well as previous studies [34] revealed that as long as puncture tests occurred within a single internode and were 2.54 centimeters (1 inch) apart and away from meristematic tissue near the nodes, they produced very similar responses.

**Fig. 5:**
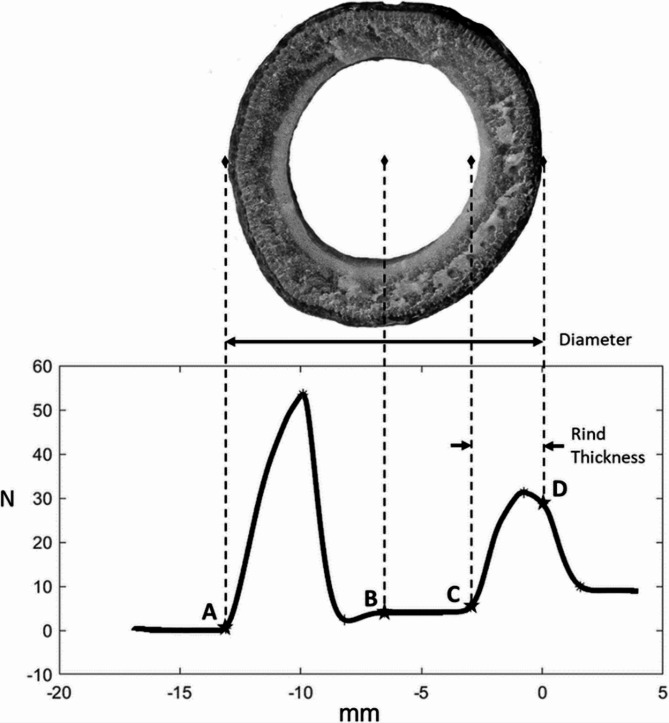
Key points of the load–extension curve from a puncture test showing how each point relates to the physical features of the stalk cross section. Labeled points are: **A**—Point of initial contact, **B**—Midpoint, **C**—Point of reengagement, **D**—Exit (zero) plane. Diameter is calculated as the distance between points **A** and **D** whereas the rind thickness is calculated as the distance between points **C** and **D** [19]

The preliminary puncture testing described above indicated that each internode could be tested multiple times at different rates. Therefore, a total of 30 maize stalk internodes were subjected to repeated puncture testing at rates of 10 mm/s, 25 mm/s and 40 mm/s. In other words, each internode was punctured three times (once at each rate). The specific puncture location within the internode was randomized according to puncture rate and only the centermost 10.16 centimeters (4 inches) of the internode were punctured. The force-displacement data from each test was processed using a custom MATLAB script as described in [35]. The MATLAB script calculated the integrative puncture score (IPS) [35], maximum force and minor diameter of the stalk at each puncture location. Figure 5 gives a brief visual example of how these values are calculated from the force-displacement data obtained from each puncture test. A more in-depth discussion is provided in [19].

## Results

### Three-point bending tests of maize stalks

#### The effect of displacement rate on flexural stiffness measurements of maize stalks

Thirty maize stalks were subjected to repeated, nondestructive bending tests at displacement rates ranging from 2.5 mm/min to 360 mm/min. All tests remained within the elastic range and no damage was observed. The flexural stiffness of the stalks ranged from 5.08 Nm^2^ to 45.81 Nm^2^. To test differences among displacement rates the flexural stiffness data was normalized. In particular, each flexural stiffness measurement of each stalk was normalized by the flexural stiffness of that same stalk obtained at a rate of 100 mm/min.

**Fig. 6:**
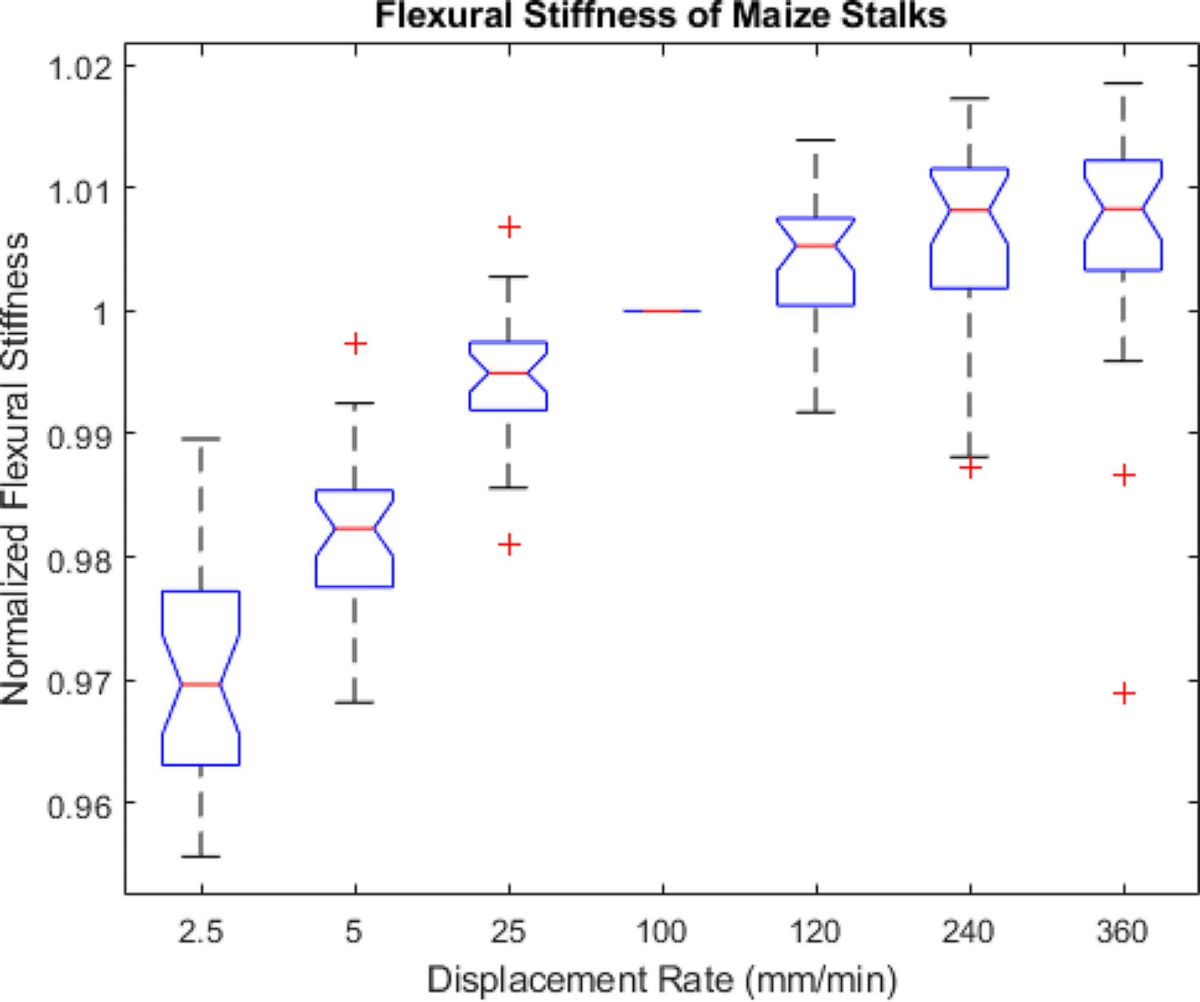
Boxplots of normalized flexural stiffness values of 30 maize stalk samples. The flexural stiffness data was normalized by the flexural stiffness value obtained at a displacement rate of 100 mm/min. The central mark in each box indicates the median, while the top and bottom edges of the box indicate the 75th and 25th percentiles, respectively. The whiskers extend to the most extreme data points not considered outliers. All outliers are plotted individually using the ‘+’ marker symbol. Notches indicate statistical significance. In particular, if the notches of two box plots do not overlap, you can conclude with 95% confidence that medians are significantly different

Fig. 6 displays boxplots of the normalized flexural stiffness data. It can be seen in Fig. 6 that displacement rate has a small but statistically significant effect on measurements of flexural stiffness. In particular, there is approximately a 4% difference in flexural stiffness observed on average between the slowest displacement rate of 2.5 mm/min and the fastest rate of 360 mm/min.

#### The effect of displacement rate on bending strength measurements of maize stalks

**Fig. 7:**
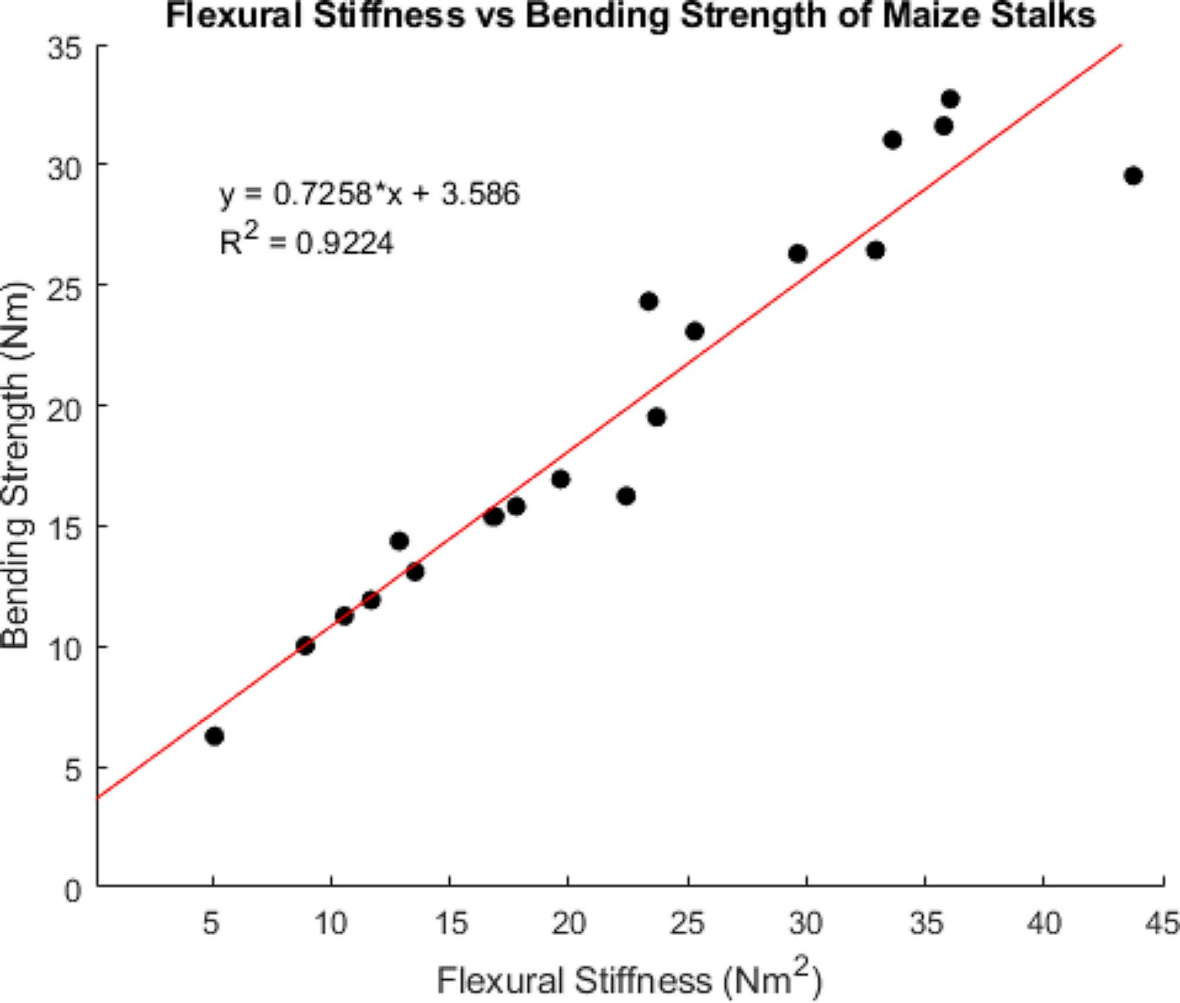
Scatter plot demonstrating the strong linear relationship between stalk flexural stiffness and stalk bending strength in maize. Measurements were acquired at a displacement rate of 100 mm/min

Eighty maize stalks were subjected to repeated, nondestructive bending tests at a displacement rate of 100 mm/min to determine flexural stiffness. The stalks were then split into four groups of twenty stalks each. Each group of stalks was tested to failure at a unique displacement rate to obtain bending strength measurements. The displacement rate of the destructive tests was 2.5 mm/min, 25 mm/min, 100 mm/min or 360 mm/min. The bending strength of the stalks ranged from 6.01 Nm to 49.39 Nm. A strong relationship between flexural stiffness and bending strength was verified for the displacement rate of 100 mm/min as seen in Fig. 7 and described in [16]. The linear regression equation between flexural stiffness and bending strength shown in Fig. 7 was therefore used to predict the bending strength of all 80 of the maize stalks at a rate of 100 mm/min.

**Fig. 8:**
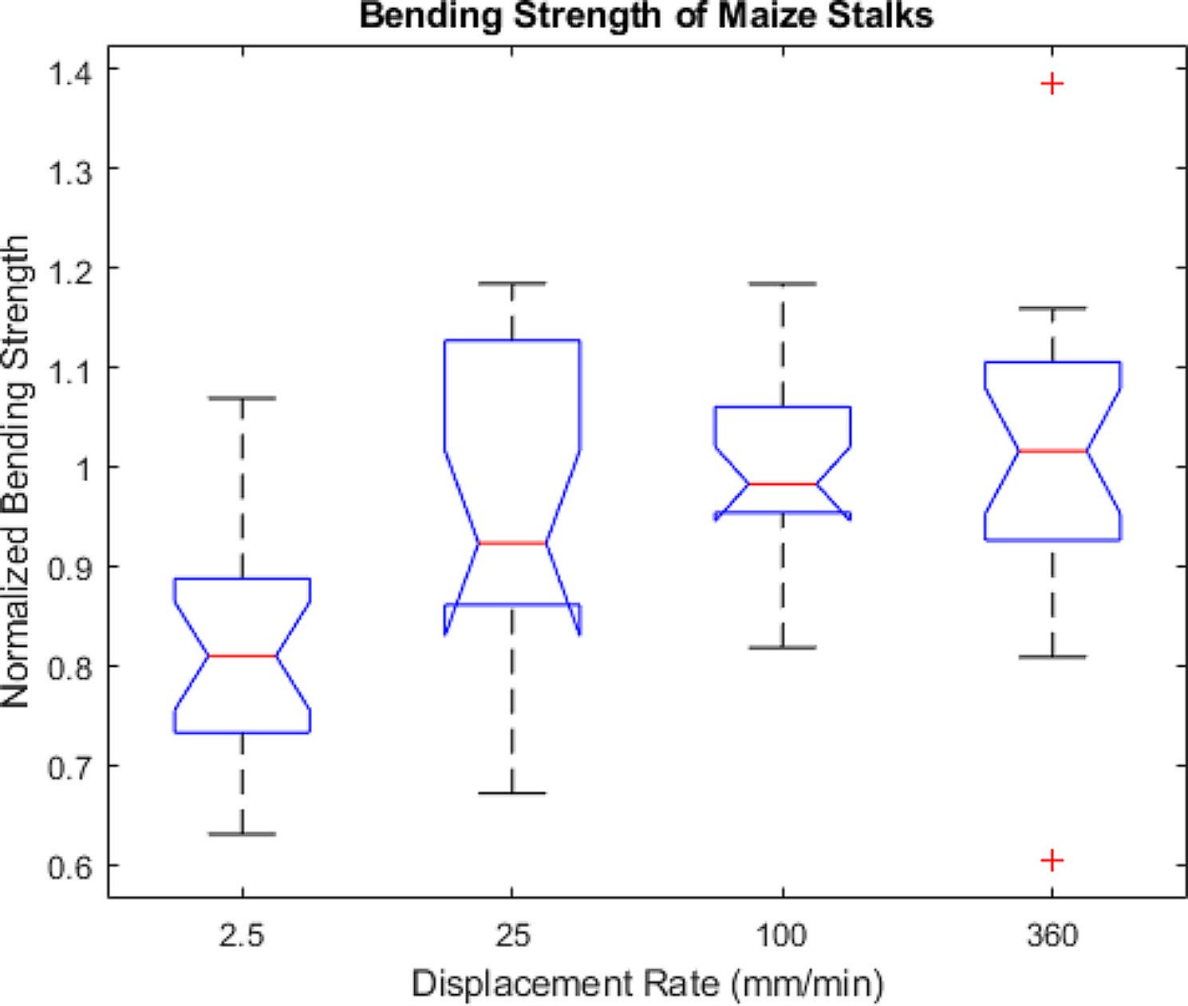
Box plots of the measured bending strength normalized by predicted bending strength by rate. Predicted bending strength was calculated using the linear relationship between flexural stiffness and bending strength shown in Fig. 7 which was obtained at a displacement rate of 100 mm/min. The central mark in each box indicates the median, while the top and bottom edges of the box indicate the 75th and 25th percentiles, respectively. The whiskers extend to the most extreme data points not considered outliers. All outliers are plotted individually using the ‘+’ marker symbol. Notches indicate statistical significance. The notches on the 2.5 mm/min boxplot do not overlap with a normalized strength value of 1. Therefore, it can be concluded with 95% confidence that the testing rate has a significant effect on bending strength measurements. However, we were unable to confirm a significant effect at the other three displacement rates shown in the figure

To determine the effect of displacement rate on bending strength all bending strength measurements were normalized. In particular, the measured bending strength value of each stalk was normalized by the predicted bending strength of the same stalk obtained at a rate of 100 mm/min. Figure 8 displays normalized boxplots of bending strength according to displacement rate. As displacement rate increased the bending strength increased with an average 20.6% increase in bending strength observed between the slowest and fastest rates.

### Three-point bending of wheat stems

#### The effect of displacement rate on flexural stiffness measurements of wheat stems

A total of 60 wheat stems (30 with leaf sheaths and 30 without leaf sheaths) were subjected to repeated, nondestructive three-point bending tests at displacement rates ranging from 1 mm/min to 144 mm/min. Flexural stiffness results obtained at 144 mm/min were removed from the study as significant inertial effects were observed during testing (i.e., the stalks were bouncing and moving on the fixture during the test) which produced highly variable data. In other words, flexural stiffness could not be measured reliably at that displacement rate with standard testing equipment. All tests remained within the elastic range and no damage was observed during testing. The flexural stiffness of the wheat stems with a leaf sheath ranged from 0.0482 Nm^2^ to 0.248 Nm^2^ and wheat stems without a leaf sheath ranged from 0.0369 Nm^2^ to 0.228 Nm^2^. To test for differences among displacement rates the flexural stiffness data was normalized. In particular, each flexural stiffness measurement of each stem was normalized by the flexural stiffness of that same stem obtained at a rate of 40 mm/min.

**Fig. 9:**
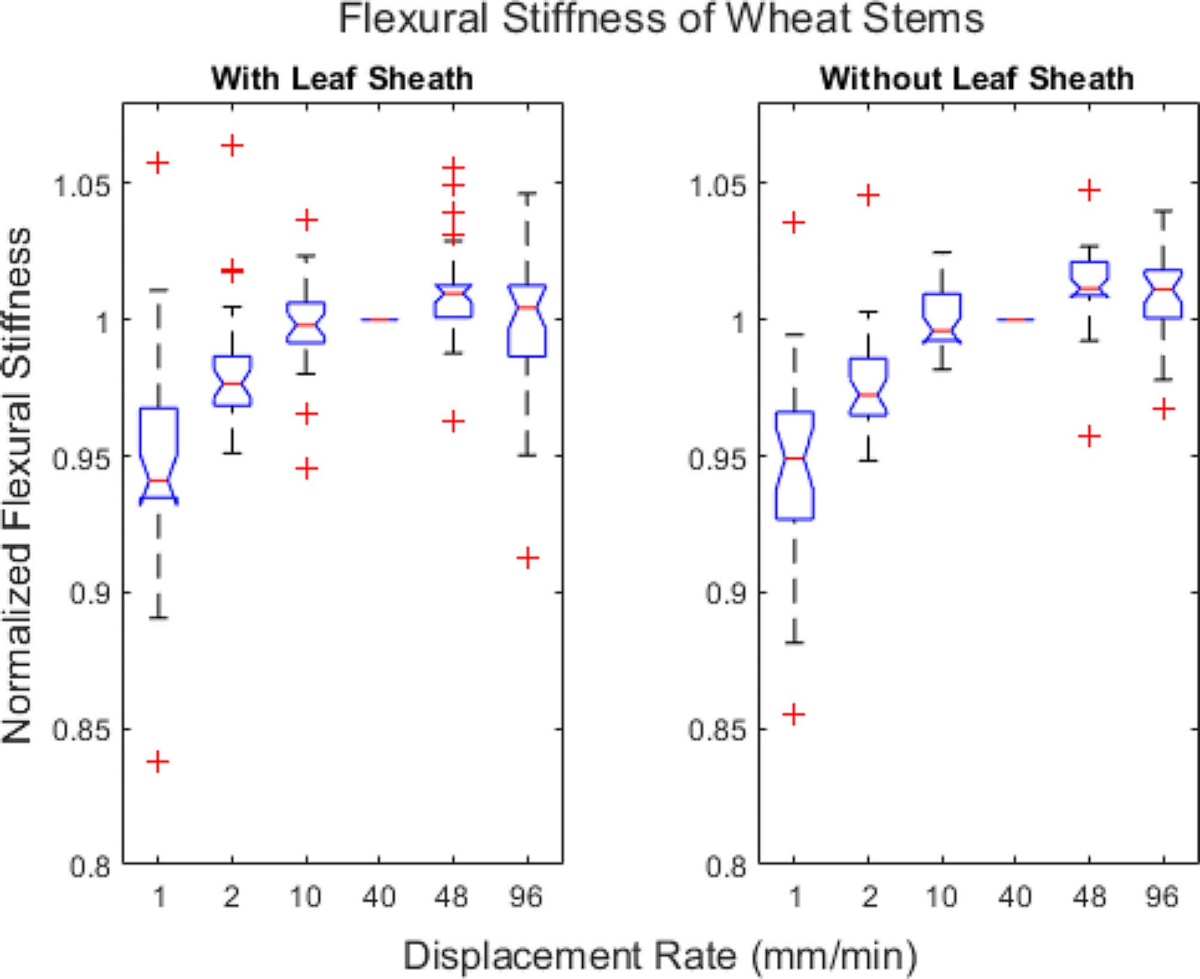
Boxplots of normalized flexural stiffness values of 60 wheat stem samples (30 with their leaf sheaths and 30 without a leaf sheath). The flexural stiffness data was normalized by the flexural stiffness value obtained at a displacement rate of 40 mm/min. The central mark in each box indicates the median, while the top and bottom edges of the box indicate the 75th and 25th percentiles, respectively. The whiskers extend to the most extreme data points not considered outliers. All outliers are plotted individually using the ‘+’ marker symbol. Notches indicate statistical significance. In particular, if the notches of two box plots do not overlap, you can conclude with 95% confidence that medians are significantly different

Fig. 9 displays boxplots of the normalized flexural stiffness data. It can be seen in Fig. 9 that displacement rate has a small but statistically significant effect on measurements of flexural stiffness. In particular, a 7% difference in flexural stiffness is observed on average between the slowest displacement rate of 1 mm/min and the fastest rate of 96 mm/min for stems with a leaf sheath. A similar 7% difference in flexural stiffness is observed on average for stems without a leaf sheath.

#### The effect of displacement rate on bending strength measurements of wheat stems

**Fig. 10:**
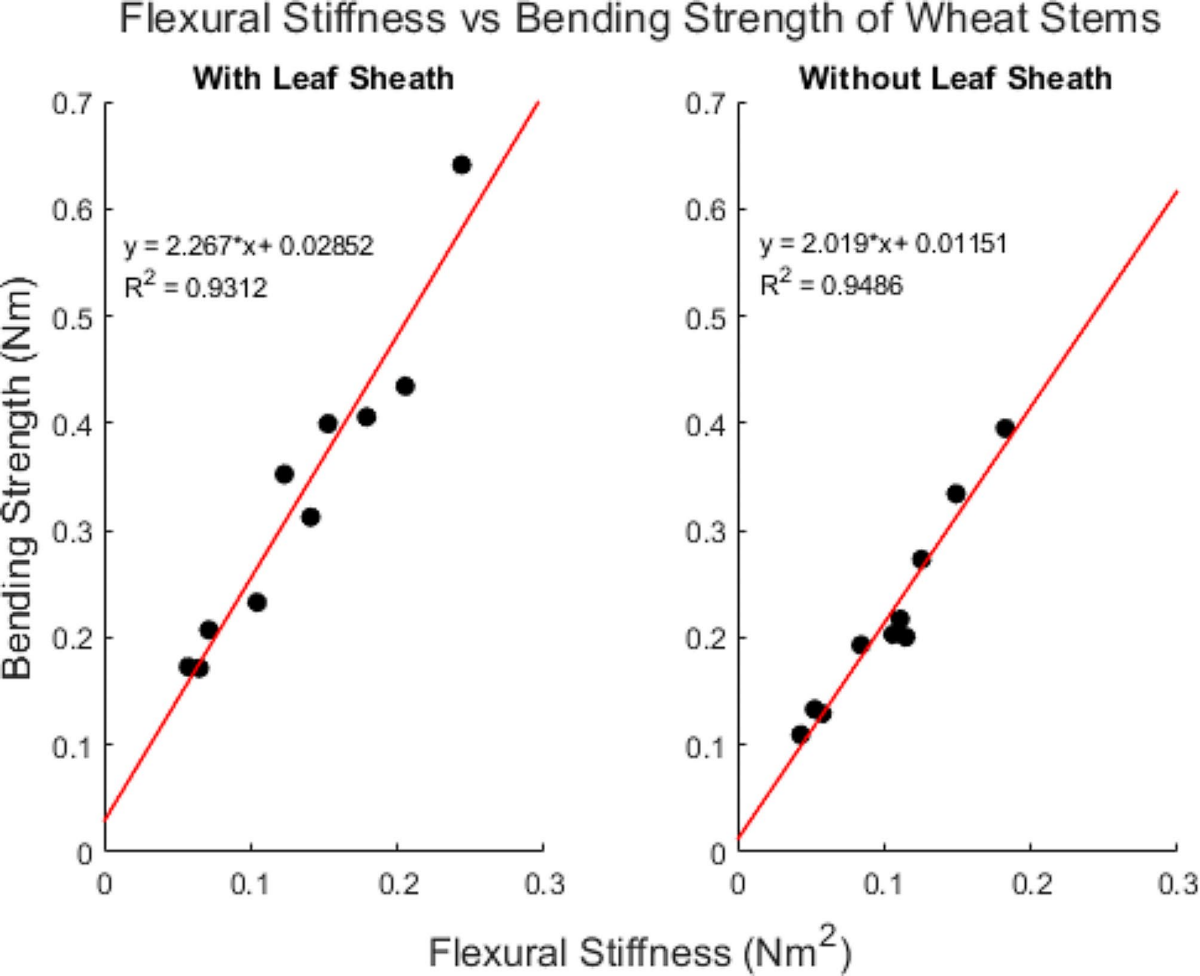
Scatter plot demonstrating the strong linear relationship between stem flexural stiffness and stem bending strength in wheat. Measurements were acquired at a displacement rate of 40 mm/min

Sixty wheat stems (30 with a leaf sheath and 30 without a leaf sheath) were subjected to repeated, nondestructive three-point bending tests at a displacement rate of 40 mm/min to determine flexural stiffness (see Eq. 1). The stems were then split into three groups of 20 stems each (10 stems with a leaf sheath and 10 stems without a leaf sheath in each group). Each group of twenty stalks were then subjected to destructive bending tests at a displacement rate of 1 mm/min, 40 mm/min or 144 mm/min. The bending strength (see Eq. 2) of stems with a leaf sheath ranged from 0.1352 Nm to 0.641 Nm and the bending strength of stems without a leaf sheath ranged 0.094 Nm to 0.4456 Nm. As expected, linear regression analysis revealed a strong linear relationship between flexural stiffness and bending strength. Figure 10 shows the linear correlation analysis between flexural stiffness and bending strength for the group of stems that were displaced at rate of 40 mm/min during both the flexural stiffness and bending strength tests.

**Fig. 11:**
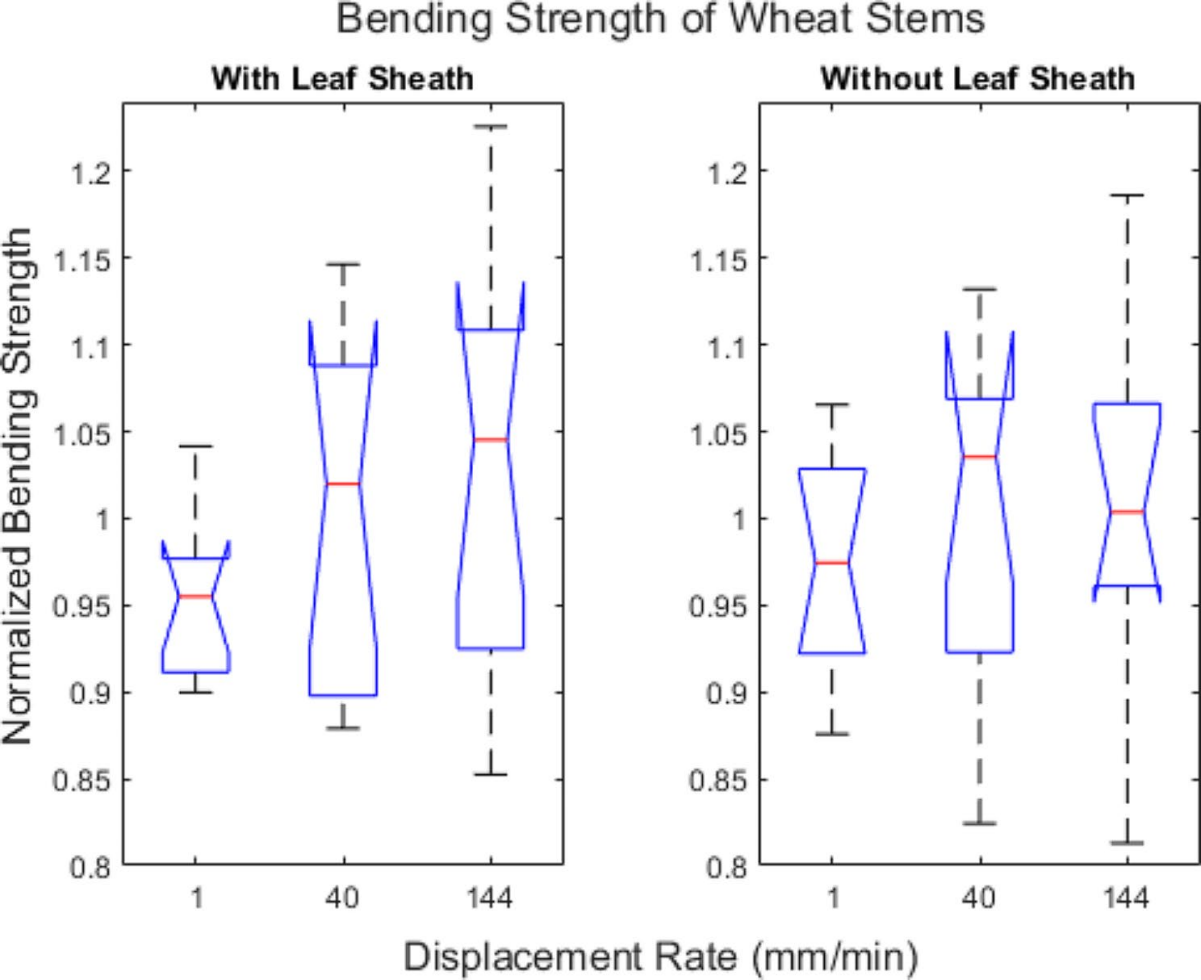
Box plots of the measured bending strength normalized by predicted bending strength stratified by displacement rate. Predicted bending strength was calculated using the linear relationship between flexural stiffness and bending strength shown in Fig. 10 which was obtained at a displacement rate of 40 mm/min. The central mark in each box indicates the median, while the top and bottom edges of the box indicate the 75th and 25th percentiles, respectively. The whiskers extend to the most extreme data points not considered outliers. All outliers are plotted individually using the ‘+’ marker symbol. Notches indicate statistical significance. The notches on the 1 mm/min boxplot with the leaf sheath do not overlap with a normalized strength value of 1. Therefore, it can be concluded with 95% confidence that the testing rate has a significant effect on those bending strength measurements. However, we were unable to confirm a significant effect of testing rate on bending strength in any of the other groups

To investigate the effects of displacement rate on bending strength all bending strength measurements were normalized. In particular, the measured bending strength value of each stem was normalized by the predicted bending strength (at a rate of 40 mm/min) of the same stem. The predicted bending strength (at a rate of 40 mm/min) was calculated using the regression equations shown in Fig. 10 and the measured flexural stiffness of that stem that was also obtained at the displacement rate of 40 mm/min. Boxplots of normalized bending strength are shown in Fig. 11. The only group that was statistically different than a normalized strength value of 1 was the group of stems tested with leaf sheath intact at a rate of 1 mm/min. In other words, we were generally not able to detect a strong influence of testing rate on bending strength measurements in wheat stems.

### Rind puncture of maize stalks

#### The effect of displacement rate on rind puncture measurements of maize stalks

To understand the effect of displacement rate on rind puncture measurements, 30 maize internodes were punctured a total of three times. Within each internode the stalk was punctured at the displacement rates of 10 mm/s, 25 mm/s and 40 mm/s. Maximum force, minor diameter and integrative puncture score measurements were obtained from each test. Maximum force measurements ranged from 25 N to 204 N, minor diameter measurements ranged from 13 mm to 25 mm and integrative puncture score ranged from 3985 N^− 1^mm^− 3^ to 269,859 N^− 1^mm^− 3^.

**Fig. 12:**
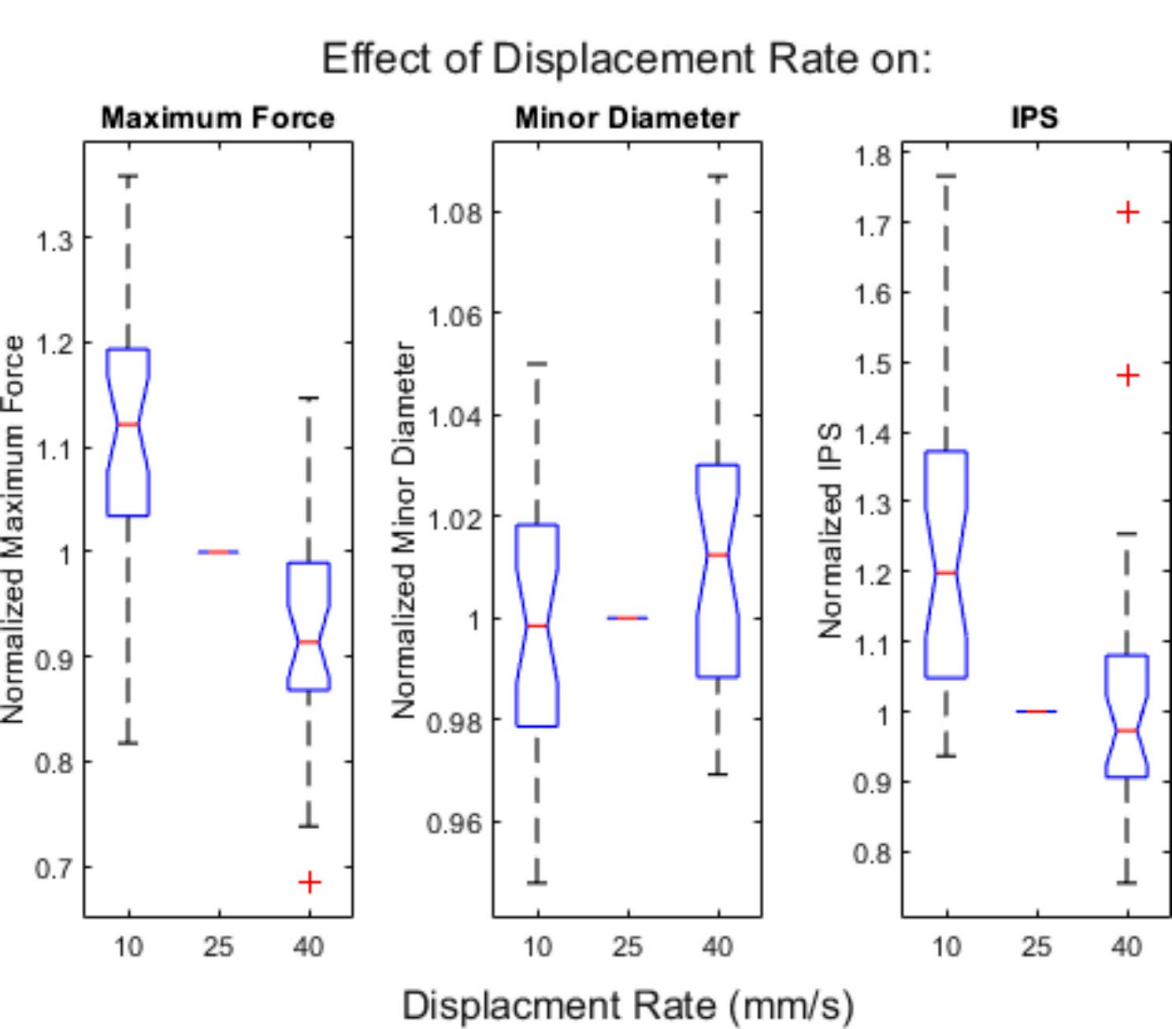
Effect of puncture rate on maximum force, minor diameter and integrative puncture score. The data was normalized by measurements obtained at a rate of 25 mm/s. Box plots of normalized maximum force, normalized minor diameter and normalized integrative puncture score by displacement rate. Each measurement was normalized by its respective “medium” rate. The central mark in each box indicates the median, while the top and bottom edges of the box indicate the 75th and 25th percentiles, respectively. The whiskers extend to the most extreme data points not considered outliers. All outliers are plotted individually using the ‘+’ marker symbol. Notches indicate statistical significance

To test for the influence of displacement rate on rind puncture measurements each measurement was normalized by the measurement obtained at a displacement rate of 25 mm/s. Boxplots of the normalized measurements are shown in Fig. 12. Maximum force and integrative puncture score measurements demonstrated statistical differences between all three displacement rates. On average, maximum force had a 20% decrease and integrative puncture score had a 22.5% decrease from a displacement rate of 10 mm/s to a displacement rate of 40 mm/s. Minor diameter did not show a noticeable statistical difference between rates.

## Discussion

### The effect of displacement rate on flexural stiffness in maize stalks & wheat stems

A 4% difference in flexural stiffness measurements of maize was observed between the displacement rates of 2.5 mm/min and 360 mm/min. In wheat a 7% difference in flexural stiffness was observed between the displacement rates of 1 mm/min and 96 mm/min. While significant, these effects are small considering the wide range of displacement rates employed and the large amount of natural variability observed in flexural stiffness data for plant stems. This data suggests testing rate has a measurable, albeit small effect on biomechanical measurements of fully mature and dry plant stems. Consequently, best practice would be to use a constant displacement rate within a study. Ideally, the displacement rate should be precisely controlled by a machine. However, it should also be possible to collect flexural stiffness data on plant stems using hand operated test stands if the users are intentional about trying to maintain the same displacement rate across tests. Hand operated tests will naturally exhibit more experimental noise as the testing rate cannot be controlled as precisely as in a computer-controlled test. Hand operated studies may therefore require larger sample sizes and averaging of collected data. However, hand operated test stands also offer several advantages including lower cost and the ability to conduct tests in outdoor or field settings. This can be particularly useful when testing crop stems. It should be emphasized that these results and suggestions apply to dry plant stems tested at displacement rates similar to what a plant would experience in its natural environment. Green stems are expected to exhibit stronger rate dependent effects and it may not be appropriate to test green stems using hand operated machinery in which the displacement rate is controlled by a human operator. Additional research is needed to determine if hand operated tests of green plant stems are sufficiently accurate.

### The effect of displacement rate on bending strength in maize stalks & wheat stems

Results demonstrated that displacement rate appears to effect on bending strength measurements of dry plant stems and stalks. However, the effect of testing rate was only statistically significant when testing at extremely slow displacement rates such as 2.5 mm/min in maize and 1 mm/min in wheat. These rates are much slower than the rates at which a plant would be deflected during a lodging event (i.e., a strong windstorm). Therefore, it appears to be feasible to conduct bending strength measurements using a hand operated test stand. When doing so care should be taken by the user to try to test all plant samples at a similar rate.

### The effect of displacement rate on rind puncture measurements in maize stalks

Results demonstrated that as displacement rate increased maximum puncture force decreased. This was unexpected. Most biological materials produce higher forces at higher displacement rates due to viscoelastic damping. We believe this trend may be explained by macro level effects including differences in cross-sectional deformation between displacement rates. In particular, we noticed that at slower displacement rates the cross-section of the stalk deforms slightly when the probe contacts the top of the stalk. The deformation observed at slower rates appears to cause the rind to pinch the probe as it is inserted. In other words, as the cross-section deforms compressive stresses are induced in the top surface of the stalk which in turn introduce increased frictional forces between the probe and stalk rind resulting in a greater puncture force measurement. At higher displacement rates the stalk cross-section does not deform and the frictional forces between the probe and stalk rind are believed to be minimal. The integrative puncture score is directly dependent on puncture force. Therefore, it follows that integrative puncture score also decreased as displacement rate increased. Minor diameter measurements did not have a statistically detectable difference between different rind puncture displacement rates. This was expected and any differences observed in minor diameter measurements are due to small difference geometry along the length of a single internode.

Rind puncture tests are generally performed in the field using a handheld force gauge and not a computer controlled universal testing system. It is very difficult to control displacement rate when conducting a hand operated puncture test. As shown in Fig. 5 the puncture force varies in a significant nonlinear fashion throughout the test. Thus, when one first presses the probe against the stalk the displacement rate is nearly zero. The user then typically continues to push harder and harder until the probe pierces the stalk. At this point the force drops dramatically, and the displacement rate therefore rapidly increases as the probe lurches through the stalk. Thus, we expect that rind puncture results would vary between a typical hand operated puncture test and the spring-loaded rind puncture instrument presented by [47]. The spring-loaded rind puncture instrument may result in more consistent data as the stalk is less likely to pinch the probe as it passes through the stalk. However, when using a spring-loaded instrument there is also a large impact force that occurs when the probe first contacts the stalk that could add noise to the measurement. Finally, it was previously shown that the strength of the correlation between hand operated rind puncture tests and stalk bending strength was similar to the strength of the correlation between machine operated rind puncture tests and stalk bending strength [34]. In summary, it appears that the hand operated, machine operated and spring-loaded rind puncture tests will all produce different results as they employ different displacement rates. Thus when comparing results across or within studies it is important to verify that the same technique was employed to gather the rind puncture data and that the same probe geometry was utilized in all of the tests [34].

### Limitations – plant materials

Plant materials utilized in this study were fully mature, senesced and dry. Results presented herein should not be extended to include the rate dependent response of hydrated or green plant materials. In general, biological materials with higher water content display more pronounced rate dependent effects as compared to materials with lower water content.

### Limitations - strain rate vs. displacement rate

In engineering literature, it is common to report strain rate as opposed to displacement rate when characterizing rate dependent material properties. However, such studies typically employ tension or compression testing regimes in which the sample geometry is strictly controlled, and the strain and stress is nearly constant throughout the test region of the sample. Tension or compression testing of plant stalks and stems presents several technical challenges and is therefore not very common. In three-point bending the strain varies throughout the sample. The maximum strain, ε, in a three-point bending test can be calculated from Eq. 3 [48] and the strain rate in three point bending can be calculated using Eq. 4.

3 $$\:\epsilon\:=\frac{12\delta\:c}{{L}^{2}}$$

4 $$\:{\epsilon\:}_{rate}=\frac{{\epsilon\:}_{2}-{\epsilon\:}_{1}}{{t}_{2}-{t}_{1}}$$

where $\:\delta\:$ is the displacement, *L* is the span width, *c* is the minor radius of the specimen and *t* is time. Thus, in three-point bending the strain rate is dependent upon both the displacement rate and the diameter of the plant stem. To subject all test specimens within a group to the same strain rate, the geometry of each specimen would need to be known, a set span length would need to be chosen and the displacement rate would have to be varied for each specimen. It is time consuming to change the displacement rate for every tested sample and therefore this is not commonly done in biomechanical plant studies where high throughput methodologies are favored. Changing the displacement rate for each sample is also problematic as inertial effects are dependent upon displacement rate and not strain rate. Thus, even if all stalks within a group were subjected to the same strain rate inertial effects would not be constant across all stalks. For these reasons the authors chose to control and present results in terms of displacement rate as opposed to strain rate. However, strain rates were calculated and are presented below for completeness.

**Fig. 13:**
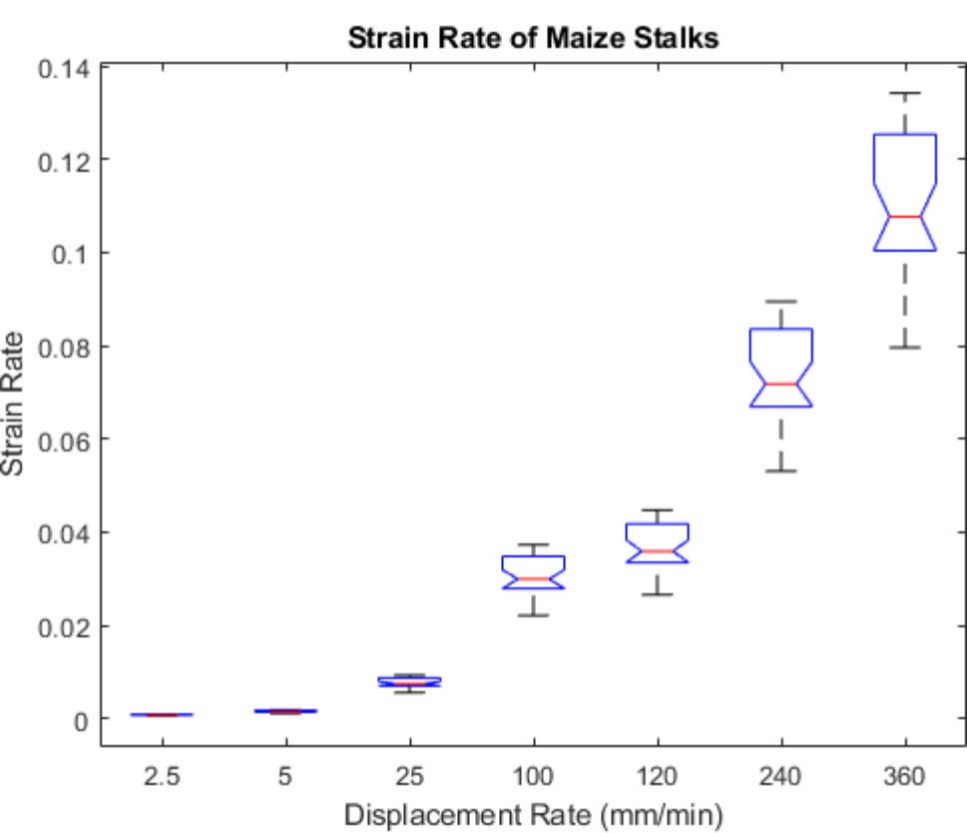
Displacement rate versus strain rate in dried maize stalks

**Fig. 14:**
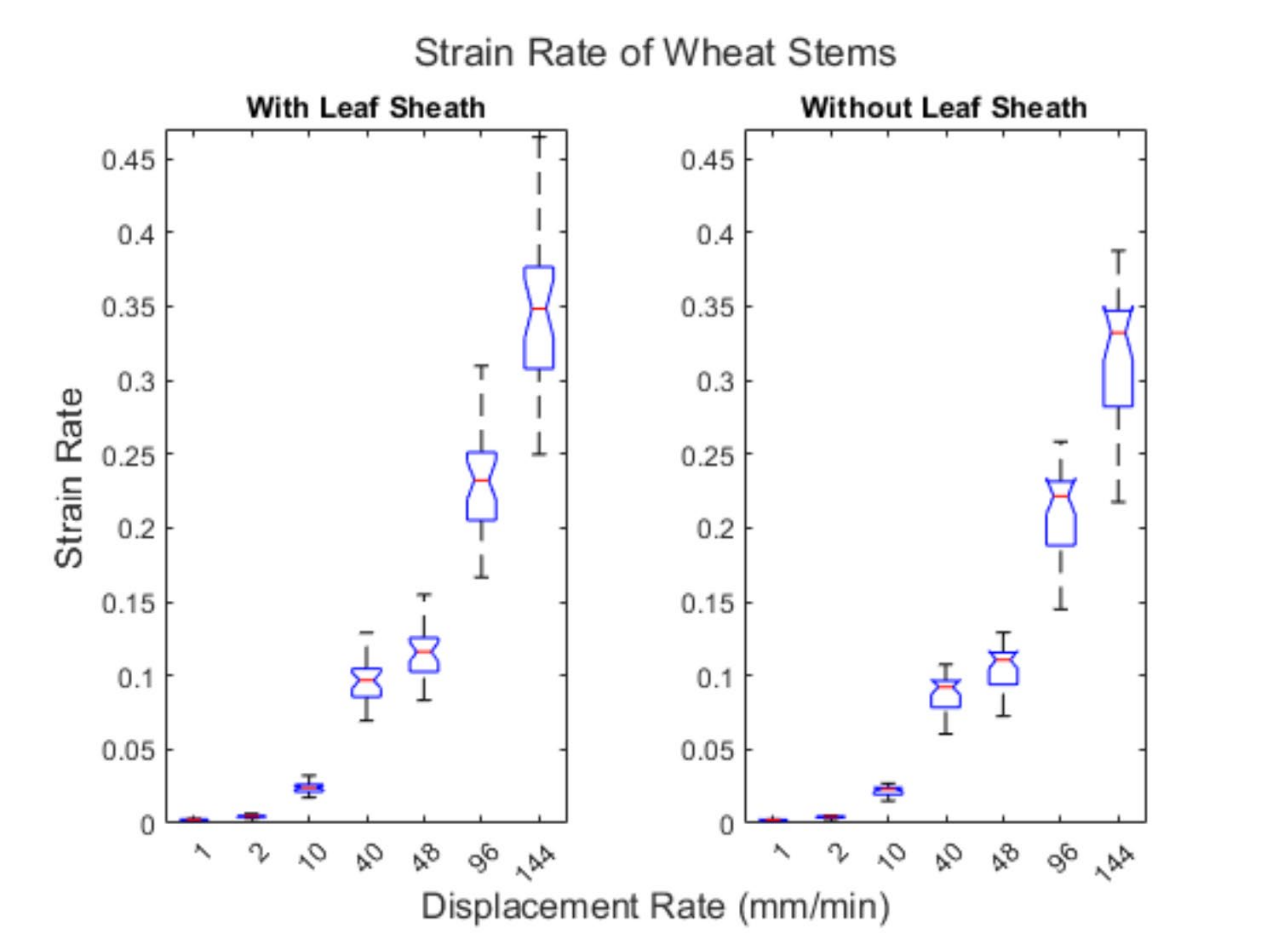
Displacement rate versus strain rate in wheat stems with and without the leaf sheath

Figure 13 displays box plots of the variance in strain rate at each displacement rate utilized when conducting the non-destructive three-point bending tests of maize stalks presented earlier. Table 3 displays the average strain rate of maize stalks at each of the specified displacement rates. Similarly Fig. 14 displays box plots of the variance in strain rate at each displacement rate utilized when conducting the three-point bending tests of wheat specimens. Table 4 displays the average strain rate experienced by wheat samples at each displacement rate. It can be seen in Figs. 13 and 14 that there is a slight overlap in strain rate between groups tested at different dispalcement rates. For example, when testing maize stalks at a rate 360 mm/min some of the smallest diameter specimens had a strain rate that was less than some of the largest diameter specimens tested at a rate of 240 mm/min. This overlap between groups is not expected to significantly impact the results of this study as paired and not grouped statistical analyses were utlized.

**Table 3:** Average strain rate of maize at the selected displacement rates and span width

Maize							
Displacement Rate (mm/min)	2.5	5	25	100	120	240	360
Average Strain Rate	0.0008	0.0015	0.0076	0.0306	0.0367	0.0734	0.1100

**Table 4:** Average strain rate of wheat at the selected displacement rates and span width

**Wheat- With Leaf Sheath**							
Displacement Rate (mm/min)	1	2	10	40	48	96	144
Average Strain Rate	0.0024	0.0048	0.0242	0.0970	0.1164	0.2327	0.3491
**Wheat- Without Leaf Sheath**							
Displacement Rate (mm/min)	1	2	10	40	48	96	144
Average Strain Rate	0.0022	0.0044	0.0220	0.0881	0.1057	0.2114	0.3171

## Conclusions

Displacement rate had a significant effect on flexural stiffness and bending strength measurements obtained from three-point bending tests of maize and wheat stems. There was a 4% difference in flexural stiffness measurements of maize and a 7% difference in flexural stiffness measurements of wheat between the slowest and fastest displacement rates. A statistical difference in bending strength measurements was only obtained when very slow displacement rates were utilized. It is recommended to conduct three-point bending tests at displacement rates similar to what a plant would experience in-vivo and to strive to keep the displacement rate uniform throughout individual future studies.

Displacement rate had a statistically significant effect on rind puncture resistance measurements. As displacement rate increased the puncture force decreased. It was hypothesized that this is due to the cross-section of the stalk deforming at slower displacement rates which induces compressive stresses in the top of the plant sample which pinch the puncturing probe.

## Data Availability

The datasets used and/or analyzed during the current study are available from the corresponding author on reasonable request.
